# Agent Orange Footprint Still Visible in Rural Areas of Central Vietnam

**DOI:** 10.1155/2014/528965

**Published:** 2014-02-03

**Authors:** Jan Banout, Ondrej Urban, Vojtech Musil, Jirina Szakova, Jiri Balik

**Affiliations:** ^1^Department of Sustainable Technologies, Faculty of Tropical AgriSciences, Czech University of Life Sciences Prague, 129 Kamycka, Suchdol, 165 21 Prague 6, Czech Republic; ^2^Dekonta J.S.C., Volutova 2525, 150 00 Prague 5, Czech Republic; ^3^Department of Agroenvironmental Chemistry and Plant Nutrition, Faculty of Agrobiology, Food and Natural Resources, Czech University of Life Sciences Prague, 129 Kamycka, Suchdol, 165 21 Prague 6, Czech Republic

## Abstract

Levels of polychlorinated dioxins/furans (PCDD/PCDF) in selected environmental samples (soils, sediments, fish, and farm animals) were analyzed from the area of Phong My commune (Thua Thien-Hue province, Vietnam). This area was affected by Agent Orange spraying during the Vietnam war (1968–1971). Whereas PCDD/PCDF content in soil and sediment samples is relatively low and ranges between 0.05 and 5.1 pg WHO-TEQ/g for soils and between 0.7 and 6.4 pg WHO-TEQ/g for sediments, the PCDD/PCDF content in poultry muscle and liver in most cases exceeded the maximum permissible limit of dioxin content per unit fat mass. In some cases of soil and sediments samples, 2,3,7,8-TCDD represented more than 90% of the total PCDD/PCDF, which indicates Agent Orange as the main source.

## 1. Introduction

The potential for TCDD contamination in Vietnam is a consequence of the war (1962–1971), during which the US Army forces used about 72 million litres of phenoxy and other herbicidal agents [1]. The spraying started in 1962 and intensified dramatically during 1967. All applications of herbicides ceased in 1971. Between 1968 and 1971, a total of 6,500 spraying missions were carried out in an area of about 1.5 million hectares, which represents about 10% of South Vietnam. Approximately one-third of the 1.5 million hectares on which the herbicide was applied was sprayed more than once and about 52,000 ha was sprayed more than 4 times. The spray was used on inland forests, cultivated soil (to destroy crops), and coastal mangrove forests. The sprays included several mixtures of defoliants; the most often used is being the so-called Agent Orange (AO), a mixture of 2,4-D (abbreviation for 2,4 dichlorophenoxy acetic acid) and 2,4,5-T (abbreviation for 2,4,5-trichlorophenoxyacetic acid). Dioxins, particularly the most toxic congener TCDD (2,3,7,8-tetrachlorodibenzo-p-dioxin), were identified as contaminants of this herbicide mixture (TCDD was formed as a by-product in the production of 2,4,5-T). The average concentration of TCDD in AO was 13 mg/kg [2]. Unlike the substances 2,4-D and 2,4,5-T that decompose relatively quickly (within months), dioxins are more stable and persist in the environment for decades. It is apparent that the concentrations of dioxins applied during the war in Vietnam have persisted in the environment until now.

The concentrations of TCDD as high as 1000 mg/kg were found in soil and sediment samples more than 30 years after Agent Orange sprays in most affected areas of Vietnam. Elevated concentrations were also identified in foods and wildlife [3]. This is evidence of long-term contamination of soil and also of the fact that a 30-year period is not sufficient for natural decontamination of these substances. On the contrary, zones of contaminations are expanding from originally contaminated zones [4]. Dioxin contamination of the food chain connected with using the herbicide Agent Orange on the territory of central Vietnam (A Luoi valley, close to a former airbase) was also reported [5].

Atmospheric deposition leads to contamination of surface water and sediments, soil, and surface of plants and thus represents a significant point of entry into the food chain. Owing to their lipophilic character, dioxins are capable of bio-accumulation in adipose tissues of animals within both terrestrial and aquatic food chains, and subsequently also of people. Moreover, several studies have demonstrated association between concentrations of dioxins in fish and molluscs and concentrations in the environment where they live [6, 7]. Experimental studies [8] have shown that dioxins in soil can accumulate in the peel of root vegetables, such as carrots. In above-ground parts of fruits and vegetables, the dominant role in deposition is played by atmospheric deposition through which lipophilic dioxins accumulate in the peel of fruits [9]. Although the transport of dioxins from soil to internal organs of plants is quite slow, accumulation of dioxins on their surfaces is, together with occasional ingestion of contaminated soil, very important source of intake of these contaminants for herbivores. It was confirmed that the dioxin level in cow milk positively correlates with the level of dioxins in their food [10].

Various adverse effects of dioxins on human health have been reported including: cancer risk, immune deficiency, reproductive and developmental abnormalities, central and peripheral nervous system pathology, endocrine disruption, decreased pulmonary functions and bronchitis, altered serum testosterone level, eyelid pathology, nausea, vomiting, loss of appetite, skin rashes, hypertrichosis, liver damage, elevated serum cholesterol and triglycerides, and enamel hypomineralization of permanent first molars in children [11–13]. An increased risk of mortality was associated with high levels of exposure to dioxins. Health consequences for Vietnam war veterans exposed to Agent Orange sprays, such as an increased risk of prostate cancer and immunotoxicological effects are frequently discussed in this context [14, 15].

In the case of Vietnam, unambiguous sources of contamination (so-called hot spots), such as former depots of chemicals and air bases, were identified and their contamination levels were precisely determined and assessed [1, 16, 17]. The objective of our research was to investigate the levels of environmental contamination by PCDD/PCDF in the area of Phong My commune (Thua-Thien-Hue province, central Vietnam). This area is not classified as a hot spot; however, it was significantly affected by AO sprays during the war and, until now, it has not been investigated.

## 2. Materials and Method

### 2.1. Site Area Description

**Figure 1:**
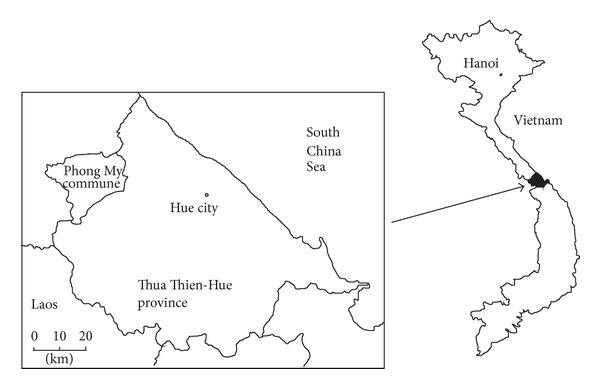
Map of Thua Thien-Hue province showing the target area of Phong My commune.

Sampling was conducted in the postconflict area of Phong My commune (Figure 1), Thua Thien-Hue province, situated in the bufferzone of Phong Dien Natural Reserve 40 km north-west of the Hue city in central Vietnam. Around 1,200 households lie in an area of 39,400 ha, circa 5000 ha of which is cultivated soil [18]. During the US-Vietnam war, the commune witnessed hard clashes in which the US Army sprayed large amounts of herbicides over the landscape. Ecological consequences of the conflict are still well apparent in the commune. Fields and hillsides are rife with craters after bomb explosions. Great parts of surrounding hills and mountains are deforested or covered with a low species-poor growth. The villages are quite large and scattered throughout the central valley of the commune. The central valley is not only a continuous settled area of villages but also of single buildings. Generally, just the central part of the valley and adjacent hillsides are inhabited. The commune consists of 10 villages and the largest, central village Luu Hien Hoa with 325 households (27% of the total commune population), was selected for this study. The annual rainfall, averaging 2,500–3,000 mm, occurs in the period from September to December. The average annual temperature is 25°C and relative humidity ranges between 85 and 88%. The principal river system in the surveyed area is Ngon O Lau River system, which originates within the Phong Dien Natural Reserve. Soils typical for this region are alluvium soils and red or yellow laterite soils. Agriculture is the main source of income for households in the whole study area. Most cultivated crops are paddy rice (*Oryza sativa* L.) and peanuts (*Arachis hypogaea* L.) especially in the central village Luu Hien Hoa [18].

### 2.2. Sampling

**Figure 2:**
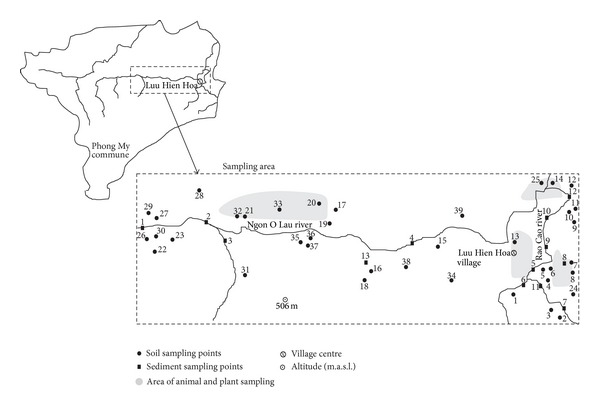
Map of sampling area in Phong My commune.

The sampling area is shown in Figure 2, where each soil sampling point represents a soil mixture taken from 5 to 10 different places in a circle of approximately 10 m, from the depth of 10–30 cm depending on local geological conditions. All the soil samples were collected using a stainless-steel soil sampler (Bűrkle, Germany). The samples were collected using vinyl gloves and a stainless-steel shovel. After collection, the soil samples were placed in appropriately labeled precleaned jars with Teflon lids and frozen. After collection, the soil samples were placed in appropriately labeled precleaned jars with Teflon lids and frozen at −20°C. The glass jars were precleaned with water, acetone, and hexane in order to prevent cross-contamination among collected samples. A total of 39 soil samples were collected from the area and 13 sediment samples from beds of ponds, reservoirs and rivers were collected in the similar way. To assess the potential impact of the pollutants on the food chain, 32 animal samples representing liver and muscle of fish, poultry and livestock were collected as well. To increase complexity of the risk analysis, four samples of commonly used vegetables and fruits were also collected and analyzed: casawa (*Manihot esculenta*)—tubers, papaya (*Carica papaya*)—fruits, banana (*Musa acuminata *×* balbisiana*)—fruits, and batata (*Ipomoea batatas*)—tubers. Before each use and between the sample collections, sampling equipment was rinsed with distiled water to avoid cross-contamination. All samples were transported frozen in a cool box (−4.0°C) to the Czech Republic for laboratory analysis.

### 2.3. Analytical Procedures

The samples were analyzed in the Czech Republic division of ALS laboratory. An ALS standard analytical procedure, according to the United States Standard EPA 1613 [19] for determination of field chlorinated dibenzo-(p)-dioxins and dibenzofurans, was used. (i) The samples were extracted with organic solvents (toluene or acetone/toluene mixture depending on the dry matter content) using ASE-300 (Dionex) or Soxhlet apparatus. (ii) The obtained raw extracts were precleaned by multiple column chromatographs in order to eliminate interference with coextracts. ALS Czech Republic laboratory provides, as the last step in the preseparation procedure, fractionation on Florisil column, where interfering chlorinated compounds, such as polychlorinated biphenyls and polychlorinated diphenyl ethers—PCDE, are separated from PCDD/F. Apart from this separation, monitoring of molecular ions of PCDE was carried out. In the case of a positive response, the fractionation was repeated, or a more suitable chromatographic column was chosen to rid the sample of interfering compounds (conformational analysis or repeated fractionation of the final extract). After that, the extracts were preconcentrated by using a rotary vacuum evaporator; the lower boiling solvents (hexane, dichloromethane) were distilled off with the modified Kuderna-Danish concentrator. Increase in the analyte concentration was accomplished through solvent evaporation, using a stream of nitrogen. Finally, for the measurement, the sample was dissolved in isooctane.

After preconcentration and spiking with the standard solution, a fraction of the final extract was analyzed by HRGC-HRMS (Double Gas chromatograph 6890N, HRMS Finnigan MAT 95 XP, and Gas chromatograph Trace GC Ultra, HRMS DFS Thermo Electron Corporation). The gas chromatograph was equipped with a TriPlus autosampler. The separation of each PCDD/PCDF was ensured by a column with polar stationary phase and the detection was carried out by a mass spectrometer working in the MID operating conditions with high resolution (*R* ≈ 10,000). Quantitative analysis was performed using selected ion current profile (SICP) areas. For identification of the analytes, the HRGC/HRMS system was calibrated and the concentration of each compound was determined by an isotope dilution technique using certified ^13^C_12_ 2,3,7,8-PCDD/F standards with response factors. Relative response factors were determined using five-point calibration for each group of the contaminants. For quantification, ALS CR used a mixture of standards which was added to the sample prior to extraction. The mixture was based on the requirements of US EPA 1613 and US EPA 1668B [19, 20].

The ALS laboratory is accredited by the Czech Accreditation Institute for a comprehensive range of analyses (testing laboratory number 1163), according to the international norm EN ISO/IEC 17025:2005. The quality of dioxin analysis at ALS laboratory was also verified by the participation in international interlaboratory studies during 2006–2009 when collected samples were analysed there (see Table 1). The published analytical results are reported in pg WHO-TEQ/g according to the toxicity equivalent factors (TEF) from 2005 [21].

## 3. Results and Discussion

The content of individual PCDD/PCDF congeners in soils and sediments is summarized in Table 2. The content of toxic (2,3,7,8 substituted) congeners of PCDD/PCDF in the collected soil samples is relatively low and ranges between 0.05 and 5.1 pg WHO-TEQ/g. The highest PCDD/PCDF content was found in samples from noncultivated areas—hillsides—above the dam, where the dioxin content was 5.1 pg WHO-TEQ/g. Average content of PCDD/PCDF in the soil samples is 2.13 pg WHO-TEQ/g and the most often found congeners are 2,3,7,8-TCDD and OCDD. Although the most toxic congener—2,3,7,8-TCDD—makes up on average 14% of the total WHO-TEQ value, in some cases, its proportion reaches up to 95%. The results of the investigation of the neighbouring district A Luoi by Hatfield Consultants (HC 1998) showed the presence of 2,3,7,8-TCDD in soil samples ranging between 86 and 94% of the total WHO-TEQ. However, the total PCDD/PCDF content in the soil samples from the area A Luoi ranging between 1.7 and 6.6 pg WHO-TEQ/g is comparable to our results. Significantly higher levels varying between 4.6 and 184 pg WHO-TEQ/g were found in the soils in the dioxin hot spot, Bien Hoa Airbase [22]. The soil samples from Phong My commune are characterized by a very low content of furans (PCDF). Overall average presence of dioxins (PCDD) in the total PCDD/PCDF content was 98% for all the soil samples.

The sediment samples originated from beds of ponds and reservoirs, where dioxins adsorbed on the mineral (argillaceous) part can theoretically be washed away during the monsoon season. In the case of sediment samples, the content of toxic congeners PCDD/PCDF is quite similar to the congeners' content found in the soil samples. Total PCDD/PCDF content in the sediments ranges between 0.7 and 6.4 pg WHO-TEQ/g. Similarly, the PCDD/PCDF levels in sediments from mangrove forests in Can Gio, South Vietnam, rural sites in Hue, central Vietnam, and from urban sites in Hanoi, the capital of Vietnam, varied between 2.7 and 9.6 pg WHO-TEQ/g [23]. The distribution of individual PCDD/PCDF congeners is similar to those of soil. The most toxic congener 2,3,7,8-TCDD makes up on average 75% of the total WHO-TEQ value in the samples, where this congener was detected. In the sediment samples the overall content of dioxins (PCDD) in total PCDD/PCDF content equals 94%.

The PCDD/PCDF content in fish muscle is summarized in Table 3. All the tested fish species originated from the dam and fishponds in Phong My commune and represented the genera *Channa*, *Cyprinus*, *Carassius*, *Clarias*, *Notopterus*, and *Wallago*. The average content of PCDD/PCDF congeners in the fish samples was 0.8 pg WHO-TEQ/g (with maximum 4.0 pg WHO-TEQ/g). According to European Directive EC number. 199/2006, the maximum permissible limit of dioxin content in fish meat and fish products is 4 pg WHO-TEQ (PCDD/PCDF/PCB) per gram of fresh weight. Except for one fish sample from the local dam, the PCDD/PCDF concentrations did not exceed the EC limit value. The highest concentrations were found in the fish species *Channa maculata* and *Clarias macrocephalus* netted in the dam; on the other hand, relatively low values were found in fish species originating from the fishponds. Average content of PCDD/PCDF in the fish adipose tissue was 37.5 pg WHO-TEQ per gram of fat. 2,3,7,8-TCDD was identified as the dominant congener (about 40% of measured value—pg/g), which suggests contamination from the Agent Orange herbicide. Similar values were also previously determined in the A Luoi valley [5] where the highest contents of PCDD/PCDF were found in the *Cyprinus carpio* sample (53.7 pg TEQ/g of fat) and the proportion of TCDD in the total WHO-TEQ value reached up to 96%. Among 13 PCDD/PCDF congeners analyzed in American Vietnam veterans compared with Vietnam era veterans, only TCDD was significantly higher leading to the identification Agent Orange as the likely source [24].

The PCDD/PCDF levels in poultry (ducks and chickens) liver and muscle (converted to pg per unit mass of fat tissue) are summarized in Table 4. In the poultry samples, the determined values of WHO-TEQ PCDD/PCDF range between 0.09 and 4.8 pg WHO-TEQ/g of sample; after conversion to pg per unit mass of fat tissue, the interval ranges from 0.4 to 15 pg WHO-TEQ/g of fat; the averages are listed in Table 4. In most cases, the determined content exceeded the limit of the European Directive EC number 199/2006 (2 pg/g of fat). Average value of WHO-TEQ PCDD/PCDF was 0.8 pg/g of sample and 4.8 pg/g of sample fat. Concerning the distribution of individual congeners, the dominant proportion of 2,3,7,8-TCDD in total WHO-TEQ is apparent. It is interesting that, unlike the fish samples, dioxin concentration in fat and muscle tissue is less variable.

In the case of hens, this phenomenon can be explained by reduced concentration of dioxins in the body as a consequence of egg production. In some cases, various contamination sources other than Agent Orange spraying, such as local combustion processes, cannot be unambiguously excluded. This fact is due to the presence of highly-chlorinated congener OCDD and also because of elevated concentrations of furans (Table 4). Since 2,3,7,8-TCDD is essentially the unique dioxin contaminant of Agent Orange, the origin of the other highly chlorinated dioxins which are not present in the Agent Orange defoliant, remains unexplained [25, 26]. Their presence in the samples of environmental matrices, as well as in human tissue, can be explained by, for example, exposure to pesticides, wood-protecting products based on chlorophenol or by combustion of organic materials, mainly those containing chlorine [27].

The Agent Orange defoliant was contaminated mainly by the 2,3,7,8-TCDD congener, formed by condensation of two molecules of 2,4,5-trichlorophenol used for production of the herbicide—2,4,5-trichlorophenoxyacetic acid (2,4,5 T). This information was used in exposure assessment of American troops in chemical units who, during the war, came into direct contact with Agent Orange. Of 13 examined congeners of dioxins and furans, only TCDD was present in increased concentrations in blood serum samples TCDD [24, 27, 28].

The PCDD/PCDF content in most of the investigated pork and beef meat samples does not exceed the limit of European Directive EC number 199/2006. In pork samples, the average PCDD/PCDF content was 0.36 pg WHO-TEQ/g, corresponding to 0.55 pg WHO-TEQ per gram of fat. In beef samples, the average content was 0.51 pg WHO-TEQ/g, which corresponds to 1.3 pg WHO-TEQ per gram of fat. According to European Directive EC number 199/2006, the limit content of dioxins in pork meat is 1 pg WHO-TEQ (PCDD/PCDF/PCB) per gram of fat. As expected, the PCDD/PCDF content in samples of vegetables and fruits is quite low. Total WHO-TEQ concentration in cassava, papaya, bananas, and batatas is 0.014 pg/g, 0.03 pg/g, 0.02 pg/g and 0.018 pg/g, respectively. No detectable content of the congener 2,3,7,8-TCDD was found in tested samples.

Even though the concentrations of dioxins in soil, sediment, and animal tissues were not significantly high (for soil and sediment the concentrations were in all cases below Vietnamese threshold values 1000 ppt TEQ or 150 ppt TEQ-TCVN 8183:2009: National Standard for Dioxins Threshold in Soil and Sediment), the cancer and noncancer risks for local inhabitants, arising from a dietary exposure (especially from fish and poultry consumption), are possible. The values of the hazard index HI for the noncarcinogenic risk of the monitored age categories ranged between 13.3 and 17.7 for maximum PCDD/PCDF concentrations in foodstuffs, while the ILCR values (lifetime increase in probability of tumour disease development) for the investigated population groups ranged between 2.8 × 10^−5^ and 1.5 × 10^−3^. This corresponds to the probability of tumour disease development approximately within the interval of 28 individuals from the group of one million inhabitants to 15 individuals from 10,000. The summary lifetime risk of tumour disease development in the monitored population corresponds to the value 2.1 × 10^−3^ for the maximum PCDD/PCDF concentrations in foodstuffs.

The assessment of spreading of the PCDD/PCDF contamination in the environment of the examined area is limited by several facts. (i) From the geological point of view, the examined area is located in a very active environment with high temperature and high rainfall, plus extensive deforestation and poor forest management. The top layer of soil is repeatedly transferred and flushed down to the valley, where it accumulates in local depressions (dams, lakes, and streams). A part of the material is transported directly to the sea. (ii) In spite of the considerable persistence of the examined substances, their decomposition is accelerated by long-lasting high temperatures and repeated transport. During the transport, intensive turbulence helps dissolution of large amounts of various substances and thus accelerates chemical decomposition of PCDD/PCDF. (iii) The area where the investigation of the contamination was performed is quite large and did not allow detailed examination through a regular network of sampling spots. Thus, we cannot exclude existence of sites with a higher degree of contamination than was revealed during the investigation. (iv) Even though rice constitutes a substantial part of the local inhabitants' diet, the PCDD/F content in rice was not analyzed within our investigation of the food chain components. Similarly, some relatively often consumed species of aquatic plants (e.g., *Ipomoea aquatica*) were not analyzed.

Clearly, there are no immediate remedies to eliminate the possibility of PCDD/PCDF exposure to inhabitants of the region. Nor is the contamination encountered at Phong My commune likely to be unique in defoliated areas of Vietnam. Such measures, which usually depend on removing PCDD/PCDF from the environment, are expensive, and contamination hotspots are therefore dealt with first. The levels of contamination in areas only hit by herbicide sprays (such as Phong My commune) are orders of magnitude lower than those in hot spots and hence it is suitable to focus on measures that prevent these compounds from entering the food chain rather than on their total elimination from the environment. Since a majority of local inhabitants consume above-average amounts of fish [29], even low concentrations of dioxins in the fish meat and fat can be hazardous for chronic dietary exposure. Because of the residual contamination of the environment, including contamination of sediments and breeding ponds, we recommend to raise fish in ponds with regularly changed and clean water free from sludge, on which PCDD/PCDF can be adsorbed. It also desirable to provide the fish with noncontaminated feed (e.g., by granules).

Similarly, for breeding of poultry, cattle, pigs, and other domestic animals, it is recommended to build a clean environment: fixed and strictly delimited areas for poultry or stables and pens for pigs. The animals should be fed only demonstrably clean feed; the rest of potentially contaminated foodstuffs (bones, entrails, skin, fat, etc.) should be excluded from the feed. Farmers should minimise the stay of cattle and domestic pigs in muddy terrain, thus preventing them from drinking strongly mudded water and minimizing contact with the sediments in natural pools, where increased contamination by PCDD/PCDF was found. Animals foraging on soil contaminated with PCDD/PCDF at low levels may bioaccumulate these compounds to unacceptable levels [10]. Since dioxins and similar substances are accumulated predominantly in animal fat, removing visible fat from meat, a preference for low-fat products, and cooking with minimum fat may lead to decreasing peoples' intake of these harmful substances. Since families in the area mostly produce their own food, their food source diversity is limited; it is therefore desirable to strive for as balanced and diverse diet as possible, with enough vegetables, fruits, and cereals. Above-ground parts of the plants can be contaminated by means of atmospheric deposition and resuspension of the soil particles and dust. Thus, the simplest way of preventing exposure from this type of contamination is careful cleaning, washing or peeling all crops in direct contact with soil or sediments or growing in a dusty environment. It is understandably beneficial to consume less produce grown in a more contaminated environment. Additionally, improper combustion of domestic refuse, plastics, paints, or painted wood produces dioxins as well should therefore be avoided. Evidently, it is necessary to regularly monitor the quality and the degree of contamination of feed used for breeding domestic and farming animals so as to prevent pollutants from getting into the muscle and fatty tissue through feed and to limit their further spreading in the environment. Local authorities should aim to reduce or prevent wastewater and sewage release into surface waters, uncontrolled landfilling, and waste incineration, as well as improper use of pesticides that contain persistent organic substances dangerous to both human health and ecosystems. Such practices significantly increase the risk of the contamination of the environment and contribute to the negative effects of PCDD/PCDF and other harmful substances. A promising development in local authority's involvement in reducing the risks of dioxin exposure at dioxin hotspots has recently been implemented in Bien Hoa City, where residents of two wards enjoy the first complex public health program based on assessment of health risks from dietary dioxin exposure [16].

## 4. Conclusions

Results of the assessment of health risks caused by exposure to dioxins in the environment (soils and sediments) and foodstuffs of the local inhabitants (liver and muscle of farm animals, fruits, and vegetables) in Phong My commune area show a potential risk from the increased PCDD/PCDF levels in the environment. Since these highly dangerous bioaccumulative and persistent substances are known carcinogens and teratogens, reducing exposure to as low as reasonably achievable is desirable. Therefore, technically and financially attainable remedial measures should be taken to reduce the exposure to these contaminants as much as possible. In addition to measures that inhabitants can take themselves, action is needed from health-care organisations, public institutions, and political authorities responsible for the health of the environment, safety of foodstuffs, quality of farm animal feed, and so forth. Raising public awareness of both the health threats arising from exposure to PCDD/PCDF and ways to minimise those threats is a key tool in dealing with the described risks. Even with fairly conservative assumptions, the ascertained health risks suggest that greater attention should be paid to the behavior and toxicity of the PCDD and PCDF group, and to exploration of exposure reduction possibilities.

## Figures and Tables

**Table 1:** Overview of international interlaboratory studies—determination of PCDD/F-TEQ in environmental matrices.

Name of study	Implementer	Lab code	Matrix	TEQ	ALS value (ng/g) or (pg/uL)	Refer. value (ng/g) or (pg/uL)	*Z*-score for TEQ*
International Interlaboratory Study IIS-01 on sludge samples, 2006	Luc Levert, M.Sc. Chimiste Chef de division Matériaux de Reference, Centre d'expertise en analyse environnementale du Québec, Canada	425	Sludge 1	Lower bound	0.0197	0.0179	NA
				Upper bound	0.0197	0.0179	
			Sludge 2	Lower bound	0.0103	0.0101	
				Upper bound	0.0103	0.0101	
			Solution	Lower bound	86.5	85.8	
				Upper bound	86.5	85.8	

International sediment exchange for tests on organic contaminants, Exchange Programme (SETOC), 2006, I.Q	SETOC-Wepal P.O. Box 8005 NL-6700 EC Wageningen The Netherlands	ECCM (841)	Sediment 1	Lower bound	0.0233	0.0216	NA
				Upper bound	0.0233	0.0216	
			Sediment 2	Lower bound	0.0430	0.0312	
				Upper bound	0.0430	0.0312	
			Sludge 3	Lower bound	0.0450	0.0424	
				Upper bound	0.0450	0.0424	
			Soil 4	Lower bound	0.0864	0.142	
				Upper bound	0.105	0.142	

11th round of the International Intercalibration Study, 2006 (in cooperation with Wepal-SETOC)	Profesor Bert van Bavel, Chairman and Coordinator of International Intercalibration Studies, Dyltabruk, Sweden	153	Ash A	Lower bound	0.179	0.151	0.74
				Upper bound	0.179	0.151	
			Ash B	Lower bound	0.343	0.333	0.32
				Upper bound	0.343	0.333	
			Ash C	Lower bound	0.196	0.192	0.21
				Upper bound	0.196	0.192	
			Sediment A	Lower bound	0.0233	0.0233	−0.03
				Upper bound	0.0233	0.0233	
			Sediment B	Lower bound	0.0356	0.0340	0.45
				Upper bound	0.0356	0.0340	
			Sludge C	Lower bound	0.0450	0.0445	−0.04
				Upper bound	0.0450	0.0445	
			Soil D	Lower bound	0.162	0.151	0.69
				Upper bound	0.162	0.151	
			Solution R	Lower bound	83.7	82.2	0.17
				Upper bound	83.7	82.2	

12th round of the International Intercalibration Study, 2007 (in cooperation with Wepal-SETOC)	Profesor Bert van Bavel, Chairman and Coordinator of International Intercalibration Studies, Dyltabruk, Sweden	153	Ash A	Lower bound	2.97	2.90	0.24
				Upper bound	2.97	2.90	
			Ash B	Lower bound	1.06	1.07	0.04
				Upper bound	1.06	1.08	
			Ash C	Lower bound	0.257	0.268	−0.18
				Upper bound	0.257	0.269	
			Sediment A	Lower bound	0.0526	0.0600	−0.94
				Upper bound	0.0526	0.0600	
			Sediment B	Lower bound	0.0229	0.0270	−0.78
				Upper bound	0.0239	0.0270	
			Sediment C	Lower bound	8.67	9.05	−0.09
				Upper bound	8.67	9.05	
			Sludge D	Lower bound	0.00192	0.0029	−0.72
				Upper bound	0.00279	0.0032	
			Solution	Lower bound	25.1	22.1	1.78
				Upper bound	25.1	22.1	

Cambridge Isotope Laboratories, 2007 International Interlaboratory Study on Fly Ash Reference Material	Jay Grazio, Study Coordinator, Cambridge Isotope Laboratories, Inc., USA	ALS Czech Rep.	Fly ash	Lower bound Upper bound	0.112 0.112	0.133 0.133	NA

13th round of the International Intercalibration Study, 2008 (in cooperation with Wepal-SETOC)	Profesor Bert van Bavel, Chairman and Coordinator of International Intercalibration Studies, Dyltabruk, Sweden	153	Ash A	Lower bound	0.162	0.165	0.08
				Upper bound	0.162	0.165	
			Ash B	Lower bound	1.01	1.00	0.25
				Upper bound	1.01	1.00	
			Ash C	Lower bound	0.186	0.167	0.50
				Upper bound	0.186	0.167	
			Sediment A	Lower bound	0.0283	0.0373	−1.27
				Upper bound	0.0285	0.0380	
			Sediment B	Lower bound	0.0360	0.0345	0.22
				Upper bound	0.0360	0.0350	
			Sediment C	Lower bound	0.726	0.723	0.17
				Upper bound	0.726	0.721	
			Sediment D	Lower bound	0.00183	0.00328	−1.03
				Upper bound	0.00312	0.00363	
			Solution	Lower bound	319	312	0.39
				Upper bound	319	312	

14th round of the International Intercalibration Study, 2009 (in cooperation with Wepal-SETOC)	Profesor Bert van Bavel, Chairman and Coordinator of International Intercalibration Studies, Dyltabruk, Sweden	153	Ash A	Lower bound	0.167	0.179	NA
				Upper bound	0.167	0.180	
			Ash B	Lower bound	0.995	0.995	
				Upper bound	0.995	0.995	
			Ash C	Lower bound	0.941	0.915	
				Upper bound	0.941	0.915	
			Sediment A	Lower bound	0.00331	0.00940	
				Upper bound	0.00385	0.00961	
			Sediment B	Lower bound	0.0664	0.0781	
				Upper bound	0.0664	0.0782	
			Sediment C	Lower bound	0.203	0.213	
				Upper bound	0.203	0.214	
			Sediment D	Lower bound	0.148	0.150	
				Upper bound	0.148	0.150	
			Solution	Lower bound	152	158	
				Upper bound	152	158	

*Acceptable *Z*-score is between: (−2.0 and +2.0); NA: *Z*-scores not available.

**Table 2:** The contents of individual PCDD/F levels in soils and sediments (pg WHO-TEQ/g); *n* = 52.

Congeners	Min	Max	Average	Standard deviation	Median	MAD
2,3,7,8-TCDD	<DL	5.50	0.449	1.06	<DL	<DL
1,2,3,7,8-PeCDD	<DL	2.80	0.069	0.407	<DL	<DL
1,2,3,4,7,8-HxCDD	<DL	0.920	0.021	0.132	<DL	<DL
1,2,3,6,7,8-HxCDD	<DL	1.40	0.042	0.204	<DL	<DL
1,2,3,7,8,9-HxCDD	<DL	2.30	0.139	0.369	<DL	<DL
1,2,3,4,6,7,8-HpCDD	<DL	3.30	0.171	0.474	0.051	0.026
OCDD	<DL	5.70	0.408	0.851	0.180	0.113
2,3,7,8-TCDF	<DL	0.036	0.001	0.005	<DL	<DL
1,2,3,7,8-PeCDF	<DL	<DL	<DL	<DL	<DL	<DL
2,3,4,7,8-PeCDF	<DL	0.083	0.002	0.012	<DL	<DL
1,2,3,4,7,8-HxCDF	<DL	0.084	0.003	0.017	<DL	<DL
1,2,3,6,7,8-HxCDF	<DL	0.084	0.002	0.012	<DL	<DL
1,2,3,7,8,9-HxCDF	<DL	<DL	<DL	<DL	<DL	<DL
2,3,4,6,7,8-HxCDF	<DL	0.055	0.002	0.009	<DL	<DL
1,2,3,4,6,7,8-HpCDF	<DL	0.280	0.016	0.041	<DL	<DL
1,2,3,4,7,8,9-HpCDF	<DL	0.005	<DL	0.001	<DL	<DL
OCDF	<DL	2.20	0.094	0.377	<DL	<DL

WHO-PCDD/F TEQ (pg/g)	<DL	24.7	1.42		0.231	

MAD: median absolute deviation; <DL: value under detection limit of the analytical method.

**Table 3:** The contents of individual PCDD/F levels in fish muscle (pg WHO-TEQ/g); *n* = 13.

Congener	Min	Max	Average	Standard deviation	Median	MAD
2,3,7,8-TCDD	<DL	1.20	0.326	0.480	0.124	0.124
1,2,3,7,8-PeCDD	<DL	0.810	0.122	0.253	<DL	0.036
1,2,3,4,7,8-HxCDD	<DL	0.084	0.014	0.027	<DL	0.007
1,2,3,6,7,8-HxCDD	<DL	0.740	0.084	0.211	<DL	0.014
1,2,3,7,8,9-HxCDD	<DL	0.081	0.009	0.023	<DL	<DL
1,2,3,4,6,7,8-HpCDD	<DL	0.100	0.017	0.033	<DL	0.002
OCDD	<DL	0.012	0.002	0.004	<DL	<DL
2,3,7,8-TCDF	<DL	0.120	0.028	0.044	0.008	0.007
1,2,3,7,8-PeCDF	<DL	0.020	0.003	0.006	<DL	0.003
2,3,4,7,8-PeCDF	<DL	0.620	0.158	0.223	0.040	0.046
1,2,3,4,7,8-HxCDF	<DL	0.054	0.008	0.016	<DL	0.002
1,2,3,6,7,8-HxCDF	<DL	0.098	0.011	0.026	<DL	0.006
1,2,3,7,8,9-HxCDF	<DL	<DL	<DL	<DL	<DL	<DL
2,3,4,6,7,8-HxCDF	<DL	0.050	0.007	0.016	<DL	<DL
1,2,3,4,6,7,8-HpCDF	<DL	0.004	0.001	0.001	<DL	<DL
1,2,3,4,7,8,9-HpCDF	<DL	<DL	<DL	<DL	<DL	<DL
OCDF	<DL	<DL	<DL	<DL	<DL	<DL

WHO-PCDD/F TEQ (pg/g fat)	<DL	3.99	0.789		0.172	

MAD: median absolute deviations, <DL: value under detection limit of the analytical method.

**Table 4:** The contents of individual PCDD/F levels in poultry liver and muscle (pg WHO-TEQ/g fat); *n* = 12.

Congener	Min	Max	Average	Standard deviation	Median	MAD
2,3,7,8-TCDD	<DL	5.60	1.75	1.79	1.10	1.10
1,2,3,7,8-PeCDD	<DL	1.800	0.596	0.720	<DL	<DL
1,2,3,4,7,8-HxCDD	<DL	0.320	0.091	0.103	0.086	0.086
1,2,3,6,7,8-HxCDD	<DL	0.730	0.248	0.220	0.190	0.099
1,2,3,7,8,9-HxCDD	<DL	0.590	0.115	0.164	0.077	0.077
1,2,3,4,6,7,8-HpCDD	<DL	0.140	0.051	0.046	0.039	0.031
OCDD	<DL	0.013	0.006	0.004	0.005	0.003
2,3,7,8-TCDF	0.120	2.30	0.567	0.624	0.280	0.130
1,2,3,7,8-PeCDF	<DL	0.210	0.057	0.055	0.041	0.025
2,3,4,7,8-PeCDF	0.290	1.90	0.805	0.477	0.720	0.330
1,2,3,4,7,8-HxCDF	<DL	0.650	0.203	0.171	0.160	0.050
1,2,3,6,7,8-HxCDF	<DL	0.700	0.185	0.180	0.140	0.079
1,2,3,7,8,9-HxCDF	<DL	0.048	0.007	0.015	<DL	<DL
2,3,4,6,7,8-HxCDF	<DL	0.330	0.087	0.094	0.072	0.072
1,2,3,4,6,7,8-HpCDF	<DL	0.050	0.010	0.015	<DL	<DL
1,2,3,4,7,8,9-HpCDF	<DL	0.009	0.001	0.002	<DL	<DL
OCDF	<DL	0.001	<DL	<DL	<DL	<DL

WHO-PCDD/F TEQ (pg/g fat)	0.41	15.4	4.80		2.91	

MAD: median absolute deviations, <DL: value under detection limit of the analytical method.
